# Supplementary material for: Individual retrotransposon integrants are differentially controlled by KZFP/KAP1-dependent histone methylation, DNA methylation and TET-mediated hydroxymethylation in naïve embryonic stem cells
Source: Epigenetics Chromatin. 2018 Feb 26;11:7. doi: 10.1186/s13072-018-0177-1 (PMC6389204; doi:10.1186/s13072-018-0177-1)
Supplement: Supplementary file 11 — Additional file 11. Pattern analysis. [file 13072_2018_177_MOESM11_ESM.zip › Patterns analysis/DataTables/extensions/Responsive/examples/styling/index.html]

Responsive examples - Styling


# Responsive example Styling

Responsive requires very little styling information of its own, with styling needed only for the
child row display when the table has been collapsed. As such, integrating Responsive with your
application should be as simple as including the Javascript and base stylesheet! This section shows
Responsive being styling using external CSS frameworks.

### Styling

- Bootstrap styling
- Foundation styling

Please refer to the DataTables documentation for full
information about its API properties and methods.  
Additionally, there are a wide range of extras and
plug-ins which extend the capabilities of
DataTables.

DataTables designed and created by SpryMedia Ltd © 2007-2014  
DataTables is licensed under the MIT license.
